# Insights on drying and precipitation dynamics of respiratory droplets from the perspective of COVID-19

**DOI:** 10.1063/5.0037360

**Published:** 2020-12-29

**Authors:** Saptarshi Basu, Prasenjit Kabi, Swetaprovo Chaudhuri, Abhishek Saha

**Affiliations:** 1 Department of Mechanical Engineering, Indian Institute of Science, Bengaluru 560012, India; 2 Institute for Aerospace Studies, University of Toronto, Toronto, Ontario M3H 5T6, Canada; 3 Department of Mechanical and Aerospace Engineering, University of California San Diego, La Jolla, California 92093, USA

## Abstract

We isolate a nano-colloidal droplet of surrogate mucosalivary fluid to gain fundamental insights into airborne nuclei’s infectivity and viral load distribution during the COVID-19 pandemic. The salt-water solution containing particles at reported viral loads is acoustically trapped in a contactless environment to emulate the drying, flow, and precipitation dynamics of real airborne droplets. Similar experiments validate observations with the surrogate fluid with samples of human saliva samples from a healthy subject. A unique feature emerges regarding the final crystallite dimension; it is always 20%–30% of the initial droplet diameter for different sizes and ambient conditions. Airborne-precipitates nearly enclose the viral load within its bulk while the substrate precipitates exhibit a high percentage (∼80–90%) of exposed virions (depending on the surface). This work demonstrates the leveraging of an inert nano-colloidal system to gain insights into an equivalent biological system.

## INTRODUCTION

Humans eject a plethora of microdroplets^1–3^ during sneezing, coughing, or even talking, which aid in rapid transport of viral loads^4^ leading to pandemics such as COVID-19.^5,6^ Such droplets remain airborne for a considerable amount of time,^7^ given the initial size and ambient conditions,^8^ and evaporate to form infective nuclei.^9^ Chaudhuri *et al.*^10^ elucidated the mechanics of respiratory droplet clouds propagating a pandemic. They utilized droplet evaporation physics and aerodynamics to derive the rate constants of the Susceptible, Exposed, Infectious, and Recovered (SEIR) model and the dominant path of transmission.^11^ Given the size distribution of respiratory droplets,^12^ the airborne desiccated nuclei have a persistent probability of assimilation via the oral or nasal passage. They might also deposit on objects of daily use to form fomites, subsequently being assimilated by a person via touch. Although the infectivity of a given droplet-nucleus/fomite is linked to the initial viral load^13,14^ as well its stability in different environments,^15–17^ it is equally important to understand the desiccation and the precipitation dynamics of the infected droplet. The general practice is to study the viral activity in cellular environments^18^ under diffusion effects^19,20^ where the precipitation dynamics are not very important. On the other hand, a droplet embodies a plethora of fluidic transport^21,22^ and couples precipitation and evaporation to the agglomeration dynamics of the virions with the cellular material to which it is attached. Dispersion of droplets^23^ in the outdoor environment can also lead these droplets to settle on external surfaces, leading to fomite based infection. Given the complexity of the experiment with actual respiratory fluid viruses, such studies have rarely been attempted.^20^ Mucosalivary fluids are known to have dissolved salts (∼1 wt. %) in addition to mucus and enzymes.^24^ Hence, in this study, we dissolved NaCl in de-ionized water at 1 wt. % as a simple surrogate liquid. Inactive nanoparticles of polystyrene (mean size 100 nm) were added to this saline solution to mimic the virions (CoV-2).^25^ Such nanoparticles have no motility akin to the virions but exhibit no rotational diffusivity either.^26^ The unique properties of a particle at the nanoscale have been examined for viral detection^27^ and drug delivery strategies,^28^ which establish their suitability in emulating the hydrodynamics of virus-laden flows over a short span of time compared to the reproductive time scales. Virus-like particles (VLPs)^29,30^ have been synthesized from inorganic material and used to avoid contamination issues involved in handling a live virus’s nucleic acid. Eventually, the current study provides insight into the location and distribution of viruses in precipitated respiratory droplets. It should be noted that while this study cannot comment on the survivability of these virions,^31,32^ the reported preferential distribution could provide key information to virologists studying the virus lifetime. Viral loads occur in the range of 10^6^ ml^−1^–10^9^ ml^−1^ of the respiratory fluid.^33^ We used a higher limit of the reported concentration to ensure adequate fluorescent intensity. The size of the particle is *d*_*p*_ = 100 nm. The mass of each particle is $m=\rho_{par}v_{par}=5.5$ × 10^−16^ g, where ρ_par_ is the density of polystyrene (1.05 g/ml). A concentration of 0.0001 wt. % would translate to ∼2 × 10^9^ of particles. However, precipitation dynamics at higher loads^34^ present a fundamental insight into nanoparticle interaction at high electrolyte concentration^35^ as well as a crucial premise for several other applications.^36^ To this end, *φ*_*np*_ would also be varied from 0.01 to 0.1 for further investigation.

## MATERIAL AND METHODS

### Sample preparation

Pure de-ionized water is used for all experiments. The salt solution is prepared by adding 1 wt. % of NaCl to de-ionized water. The criteria for selecting the nanoparticles in this study were two-fold. (1) The NPs should be of a dimension similar to that of the virus (∼100 nm) and should be biologically and chemically inert. This allowed us to separate the effect of hydrodynamics of the virus and related accumulation in drying droplets. (2) The NPs should be fluorescent when excited with green lasers commonly used with microscopes. This allowed us to identify the particle distribution in dried nuclei. Based on these two criteria, polystyrene NPs were found to be a viable candidate. 100 nm nanoparticles of latex procured from Sigma-Aldrich are added to the salt solution in various concentrations ranging from 0.1 wt. % to 0.0006 wt. %. DLVO theory predicts particle agglomeration at sufficiently high electrolytic concentrations (>0.1M) due to increased ionic screening. However, we observe the nanoparticles to be stable for the duration of the experiment. Solutions are initially stored in 2 ml centrifuge tubes to suppress the loss of solvent and are discarded after 5 h to avoid errors due to evaporation. Akiyama *et al.*^37^ analyzed the interaction between charged colloidal particles in electrolytic solutions by omitting DLVO theory and found the dispersion to be stable at large separations. This is true for the initial low values of nanoparticles reported in the current study (<0.1 wt. %).

### Shadowgraph of the levitated droplet

Acoustic levitators use standing waves to suspend objects, such as droplets. In our experiments, a droplet was generated at the tip of a needle and carefully positioned close to the pressure node of the levitator. Once the droplet was detached from the needle, it levitated in the acoustic field. Images of the evaporating droplet were acquired using a CCD camera (NR3S1, IDT) in a shadowgraph mode. The camera and an LED source (Karl Storz) were placed opposite to each other while the levitator was inserted in-between. The droplet trapped by the levitator obstructs the light from the LED source and results in a shadow-like image acquired by the camera. 10 images are acquired at a rate of 500 fps every 3.3 s for the entirety of the droplet lifetime. The images are processed using ImageJ software as follows: the background of the images is subtracted, and they are converted to binary. Using the “Analyze Particles” plugin, an ellipse is fitted to the droplet, and the effective diameter is calculated as $D=\sqrt[{3}]{d_{x}^{2}d_{y}}$, where *d*_*x*_ and *d*_*y*_ are the major and minor axis of the fitted ellipse, respectively.

### Flow visualization of the levitated droplet

860 nm latex particles (R900, Thermofisher, density 1.05 g/cc) are added as flow tracers to the DI water, salt solution, and nanoparticle seeded salt solution at an initial concentration of 0.008 wt. %. A 0.2 W laser beam (Cobolt Samba) measuring 1.1 mm in spot size is focused on the levitated droplets for less than 2 s (to avoid heating of the droplet). The laser is positioned perpendicular to a high-speed camera (Mini UX100, Lavision). The scatter from the droplet is sampled at the rate of 2000 fps. This is repeated every 2 min for the duration of the droplet lifetime [see Fig. 2(a)].

### Laser visualization of the levitated droplet

Crystallization videos are acquired at 50 fps using the same camera arrangement as described above. Images are recorded once the diameter shrinks to 0.3 times the initial value, and the recording is continued until the crystallization is complete. These experiments do not have any added particles.

## RESULTS AND DISCUSSION

### Evaporation dynamics

**FIG. 1.**
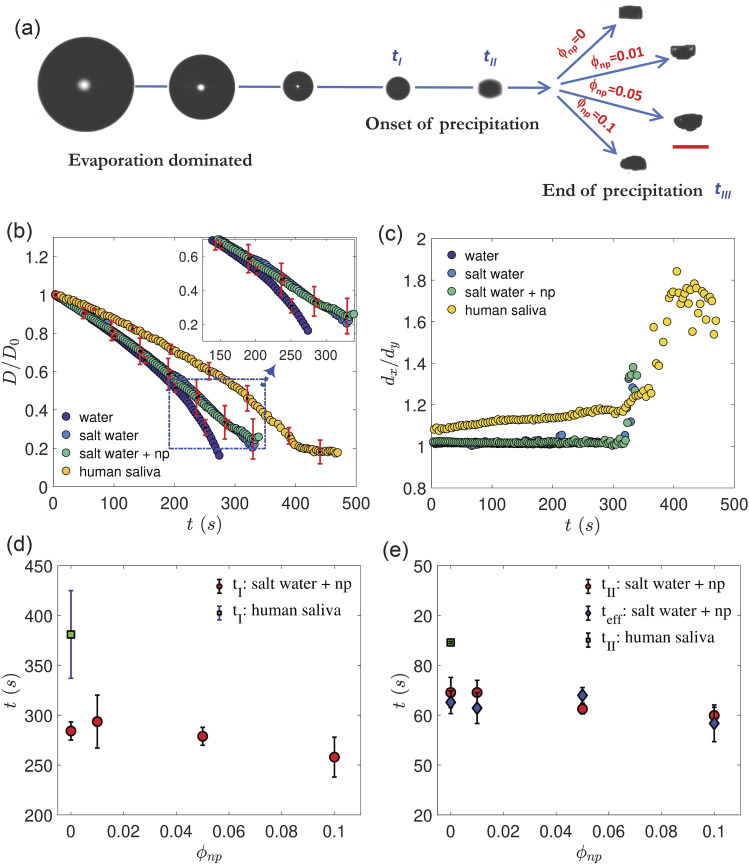
Evaporation dynamics of a levitated droplet. (a) Sequential snapshots show the reduction in the droplet diameter culminating into the final precipitate shown for different values of nanoparticle concentration (*φ*_*np*_ in wt. %). Time instant *t*_I_ indicates the end of the evaporation dominated stage when the rate of diameter reduction is significantly slower. Time instant *t*_*II*_ indicates the departure from sphericity of the droplet. The time instant *t*_*III*_ indicates the end of the process. The scale bar is 0.2 mm. (b) The droplet diameter is plotted as *D/D*_0_ vs time (t) pure water, salt-water (1 wt. %) and salt-water + nanoparticles (np), where the mean concentration of the range *φ*_*np*_ = 0.01–0.1 is used. The mean value of *D*_*0*_ = 550 ± 10 *μ*m. Error bars are standard deviations of multiple runs. (c) The aspect ratio of the droplet (*d*_*x*_*/d*_*y*_) vs *t* for the same conditions as (b) where *d*_*x*_ and *d*_*y*_ refer to the major and the minor axis of the droplet, respectively. (d) Variation in *t*_*I*_ vs *φ*_*np*_ for both surrogate and human saliva (HS). (e) Comparison of *t*_*II*_ for both surrogate and HS. The onset of efflorescence (*t*_*eff*_) for different values of *φ*_*np*_ is also plotted. The ambient temperature is set to 28 ± 0.2 °C, and the RH is set to 41 ± 2%.

Given the experimental complexity associated with studying a mobile air-borne droplet, we have used an acoustic levitator to trap a droplet in the air (tec5) and allowed it to evaporate in a controlled ambience (T_∞_ = 28 ± 0.2 °C and RH_∞_ = 41 ± 2%). Acoustic levitation^38^ has been extensively used to study the evaporation^39^ and precipitation dynamics of a solute laden droplet.^36,40–42^ A droplet of the surrogate fluid having an initial diameter *D*_0_ = 550 *μ*m + 10 *µ*m is inserted into one of the stable nodes of the acoustic levitator and imaged every 3 s at 30 fps (see Materials and Methods) until the end of evaporation. The effective diameter of the droplet $D=\sqrt[{3}]{d_{x}^{2}d_{y}}$, where *d*_*x*_ and *d*_*y*_ are the major and minor axis of the droplet, respectively. The typical lifetime of droplets with various nanoparticle concentrations is shown in Fig. 1(a). The droplet monotonically reduces until the time instant *t* = *t*_*I*_, where the shrinkage appears arrested. Subsequently, the droplet’s shape deviates from its initial sphericity (*d*_*x*_/*d*_*y*_ = 1) at *t* = *t*_*II*_ and finally assumes its crystalline form at *t* = *t*_*III*_, shown for different concentrations of nanoparticles.

The diameter reduction of four different liquids, pure water, NaCl solution (surrogate for mucosalivary fluid), NaCl solution with nanoparticles (surrogate for virus-laden mucosalivary fluid), and human saliva (HS) is plotted in Fig. 1(b). Saliva was obtained from one of the authors (healthy subject). Since the presence of nanoparticles until *φ*_*np*_ = 0.1 shows no distinctive effect on the reduction in the diameters, only the mean concentration (*φ*_*np*_ = 0.05) is plotted [Fig. 1(b)]. The initial stage of evaporation is diffusion limited^39^ and fits the standard D^2^ law which states that^43,44^ $D{\left(t\right)}^{2}=D_{0}^{2}-K_{e}t$. The value of *K*_*e*_ ∼ *O*(10^−9^) m^2^/s for pure water droplets and predicts the total lifetime to be $t_{evap}=D_{0}^{2}\;/K_{e}\approx$ 300 s, which is close to the observed values [Fig. 1(b)]. Initial droplet reduction rates are nearly equal for water and surrogate fluid but start deviating at *t* > 200 s [the inset of Fig. 1(b)] due to the presence of dissolved salt which reduces the vapor pressure of the droplet.^36^ This is consistent with the evaporation–precipitation model presented by Chaudhuri *et al.*^10^ Complex fluid droplets may have slower rates of evaporation due to the strong interaction between the solvent and the dissolved protein and other organic compounds. We have demonstrated this by drying a levitated droplet of human saliva. The (HS) droplet is shown to evaporate slower than the surrogate fluid droplets, as shown in Fig. 1(b). We note that the evaporation and precipitation time scales of human saliva droplets show about 30% difference from the surrogate liquid used (aqueous solution of 1% NaCl), which can be attributed to the variation in compositions of human saliva and the presence of additional dissolved salts and proteins of higher molecular weights. Nevertheless, phenomenological similarity of the evaporation process and the final droplet diameter after desiccation between the surrogate and real HS was observed.

It is to be noted that the experiments with droplets with other diameters (300, 600, and 800 *µ*m, shown in the supplementary material) confirmed that the nuclei of the dried droplet are about 20%–30% of the initial size and are independent of the initial droplet size.

### Precipitation dynamics

The end of the evaporation dominated phase occurs at *t* = *t*_*I*_ when the diameter shrinkage dramatically reduces, leading to a knee-like appearance [see Fig. 1(b)]. However, solvent loss, although slower, continues until *t*_*III*_. The knee-transition occurs at *t* = *t*_*I*_ = 260 s–300 s for the surrogate droplet and at *t*_*I*_ = 380 s for the HS droplet, as shown in Fig. 1(d). The knee formation is universally observed for both HS and at 0.2 ∼ 0.3*D*_*0*_, as corroborated from experiments with different initial droplet sizes (300 *μ*m–800 *µ*m), temperature ranges (27 °C–30 °C), and RH values (40%–50%) (see S1; supplementary material). The onset of the knee is independent of *φ*_*np*_ which de-couples the viral loading effect on the precipitation dynamics within the respiratory droplet. The distribution of nanoparticles within the droplet bulk can be predicted from the mass Peclet number $Pe_{m}=\frac{Ur_{0}}{D_{np}}∼$ *O*(10^2^), where the appropriate velocity scale, *U*, is the rate of diameter reduction (∼2.8 *μ*m/s), *r*_0_ is the initial radius of the droplet, and *D*_*np*_ is the mass diffusivity of nanoparticles in water calculated from the Stokes–Einstein equation $D_{np}=\frac{k_{B}T}{6\pi \mu r_{p}}∼O\left(10^{-12}\right)$ m^2^/s. For *P*_*em*_ ≫ 1, the nanoparticles do not diffuse but accumulate near the receding interface of the droplet.^35,41^

The droplet shape evolves under evaporation and departs from its initial sphericity, as shown by the plot of (*d*_*x*_/*d*_*y*_) at *t* = *t*_*II*_ [Fig. 1(c)]. The transition can be predicted as follows: the Peclet number for a levitated saline droplet^45^ is $Pe=\frac{Ur_{0}}{D_{s}}\approx$ 0.5, where *D*_*s*_ ∼ *O*(10^−9^) m^2^/s is the diffusion coefficient of NaCl in water.^46^ *Pe* < 1 indicates the homogeneous distribution of salt, allowing the use of droplet volume to estimate its bulk concentration in the droplet. At a time *t* = *t*_*eff*_ corresponding to *D/D*_*0*_ < 0.26, the efflorescence limit (640 g/l)^47^ is achieved within the bulk of the droplet. The close match between *t*_*II*_ and *t*_*eff*_ is shown in Fig. 1(e), proving the near coincidence of efflorescence and shape flattening. The HS droplet flattens at an early stage, possibly due to the naturally occurring surfactants and the acoustic pressure^48,49^ and transitions at *t*_*II,HS*_ ∼ 370 s. Thus, the evaporation and the precipitation dynamics of the surrogate droplet closely match the HS droplet based on the timescales *t*_*I*_ and *t*_*II*_. The proposed timescales are well predicted from the evaporation model,^7^ and simple bulk concentration calculations indicate the advent of crystallization in saline or HS droplets and are applicable to a wide range of droplet sizes and ambient conditions. Nanoparticle loading concentration does not appear to affect the evaporation and precipitation dynamic and thus decouples the role of viral aggregation and precipitate shape. Based on this, only the case of *φ*_*np*_ = 0 is used to discuss the role of acoustic streaming on crystallization.

### Internal flow field

**FIG. 2.**
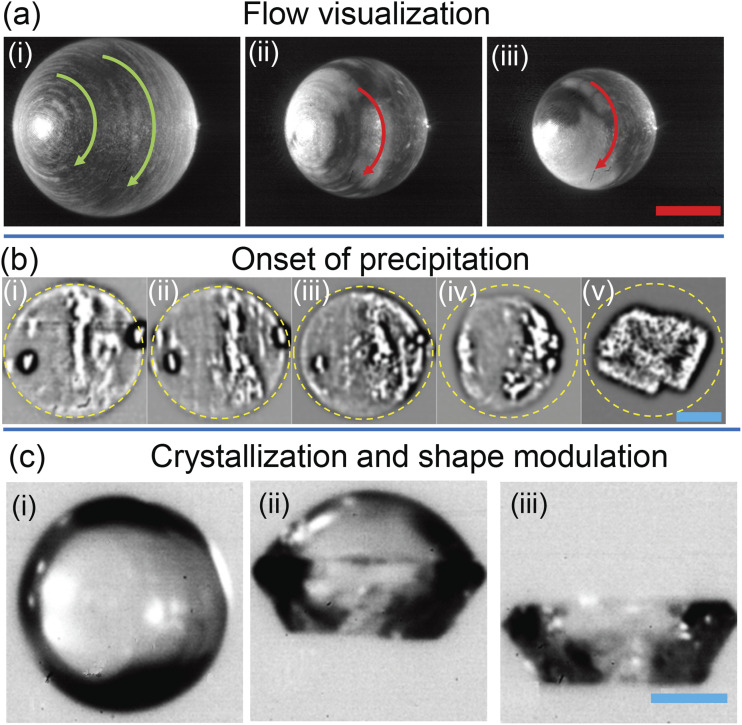
Flow visualization in case of *φ*_*np*_ = 0 is displayed as the superposition of three consecutive images (3/2000 s) for *φ*_*np*_ = 0 for (i) *D/D*_*0*_ = 1, (ii) *D/D*_*0*_ = 0.8, and (iii) *D/D*_*0*_ = 0.7. The scale bar in red is 0.2 mm. (b) The progression of precipitation in *φ*_*np*_ = 0 is laser visualized and presented at (i) *D/D*_*0*_ = 0.27, (ii) *D/D*_*0*_ = 0.26, (iii) *D/D*_*0*_ = 0.24, and (iv) *D/D*_*0*_ = 0.2 intervals and (v) the final crystalline form. (c) The front illuminated droplet shape for *φ*_*np*_ = 0 is shown at (i) *D/D*_*0*_ = 0.25, (ii) the spherical top-half and crystalline bottom half of the droplet, and (iii) the final crystalline form. The scale bar in blue is 50 *µ*m. The angle of imaging leads to the asymmetric appearance of the vortical flow.

Acoustic streaming around the droplet governs the internal flow field^38,50^ and is visualized by adding 860 nm particles of latex (1.05 g/cc) at an initial concentration of 0.008 wt. %. Illumination is carried out using a laser beam of 1 mm at 0.2 W (see S1; supplementary material). The time-averaged flow field in Fig. 2(a) shows a circulatory motion within the droplet, where a fluid particle near its surface moves at a mean rate of 0.087 ± 0.02 m/s. This homogenizes the salt molecules in the azimuthal direction (but not in the radial direction where it diffuses). The flow magnitude and direction agree with previous studies of particle image velocimetry in evaporating levitated droplets and remain nearly constant throughout the droplet lifetime,^51^ as observed from Figs. 2(a-ii) and 2(a-iii). Note that an ejected respiratory droplet is accompanied by a turbulent jet transitioning to a turbulent puff, leading to similar rotatory motions,^4^ which is recreated in this case due to the acoustic streaming and torque provided by the levitator.^38^ In this context, we would also point out that apart from very early stage of the exhalation process during respiratory events, droplets are generally considered to be in a dilute cloud, i.e., the droplets are separated by a large distance. The model presented in our previous work^10^ also confirms that the Reynolds number based on relative velocity between droplets and surrounding jets reduces to a small value very quickly. Hence, the condition for evaporation can be simulated by a single droplet experiment in the acoustic levitator.

### Onset of precipitation

**FIG. 3.**
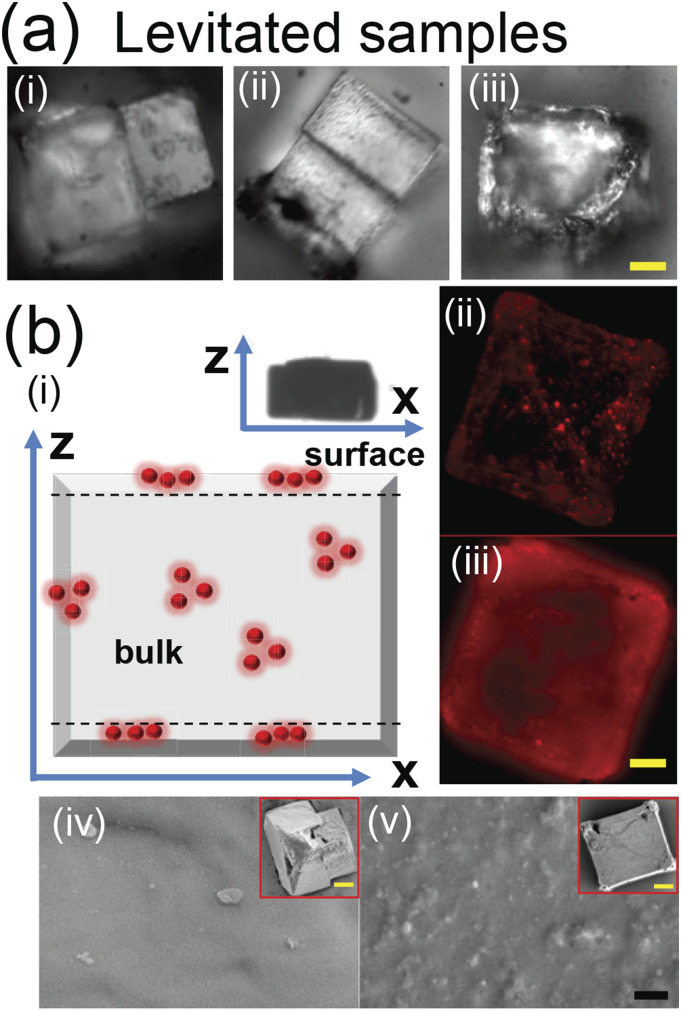
Micrograph of the preserved precipitate from levitated droplets of (i) *φ*_*np*_ = 0, (ii) *φ*_*np*_ = 0.1, and (iii) *φ*_*np*_ = 0.0001 (viral load) and (b) (i) the schematic of the levitated precipitate with viral load showing entrapped nanoparticles (red spheres). The symbol z represents the levitator axis while x represents the corresponding perpendicular direction (inset shows the final shape of the levitated precipitate). (ii) The fluorescent image of the precipitate at depths of (ii) z = 1 *µ*m (on the surface) and (iii) 21 *µ*m (within the bulk). (iv) SEM of *φ*_*np*_ = 0 (inset shows the complete precipitate under SEM). (v) The same sequence of images as (iv) for *φ*_*np*_ = 0.0001. Scale bars in yellow equal 20 *μ*m and in black equal 1 *µ*m.

Laser scattered in the absence of 860 nm particles aids in visualizing the onset of precipitation. The scatter from the droplet is sampled at a rate of 50 fps (for details, see S1; supplementary material). Images are bandpass filtered to enhance the precipitation induced scatter within the droplet. At *D*/*D*_*0*_ = 0.26–0.27, the scatter from the center of the droplet may indicate the onset of precipitation [Figs. 2(b-i) and 2(b-ii)], which coincides with efflorescence as previously discussed. At *D*/*D*_*0*_ = 0.24, the droplet interior shows uniform scatter [Fig. 2(b–iii)], while the departure from sphericity occurs at *D*/*D*_*0*_ = 0.2 [Fig. 2(b–iv)], which shows an even higher uniformity in scatter. Although spatial inception of efflorescence is difficult to identify, a drastic shape change could be observed when the bulk has crystallized, as seen from the time-lapse between Figs. 2(b–i). The final cuboidal shape of NaCl^52^ is observed from Fig. 2(b–v) at a time *t*_*III*_ = 320 s–330 s. The shape evolution is better visualized using front illumination (see S1 of the supplementary material), as shown in Fig. 2(c). The spherical shape shown in Fig. 2(c–i) transforms into a dual structure where the lower half has crystallized before the upper half [Fig. 2(c–ii)]. Saha *et al.*^34^ attributed this to an unequal pressure distribution at the north and the south poles. Consequently, the salt distribution accumulates faster in the lower half of the droplet, leading to earlier crystallization. The final cuboidal shape shown in Fig. 2(c–iii) is consistent with that of Fig. 2(b–v) but maybe different from those observed from salt precipitation in the atmosphere due to the absence of acoustic pressure field. The rate of crystal growth can be estimated as $\frac{0.3D_{0}-0.2D_{0}}{t_{III}-t_{I}}$ = 2 *μ*m/s–2.3 *μ*m/s. The final crystal dimensions are similar for various nanoparticle loadings [Fig. 3(a)].

### Viral distribution in levitated samples

The timescales of evaporation and precipitation dynamics are established in the preceding discussions. The morphological similarity between the various precipitates of different compositions (*φ*_*np*_) [Fig. 3(a)] further demonstrates the independence of precipitation from particle loading rates. To scrutinize the distribution of nanoparticles (emulated viral loading) upon precipitation, marker nanoparticles with a fluorescent label (R100, Thermofisher) are loaded into the levitated droplet at *φ*_*np*_ = 0.0001. Precipitation will entrap the nanoparticles in the levitated precipitate [Fig. 3(b–i)], similar to the entrapment of virions in desiccated airborne droplets. Here, z is along the levitator’s axis. The preserved levitated precipitate is observed in the fluorescence mode (BX51, Olympus) with a 100× objective (depth of focus ∼2.5 *μ*m) at different depths (interval of 3 *μ*m–5 *μ*m). The surface layer in Fig. 3(b–ii) shows discrete bright spots corresponding to groups of nanoparticles. The image also conveys details on sparse distribution of nanoparticles. A typical section of the precipitate bulk [Fig. 3(b–iii)] displays diffuse emission from multiple layers, possibly due to a higher concentration of particles. Using scanning electron microscopy (SEM), the surface of the same precipitate shown in Figs. 3(b-ii) and 3(b-iii) is presented, as shown in Fig. 3(b–v). Particles trapped near the upper layers of the precipitate are scanty and appear partially exposed (not stacked as multilayers), as shown in Fig. 3(b–v). In the absence of particle loading, the surface topography of the precipitate is very smooth, as shown in Fig. 3(b–iv). Thus, loaded particles/virions in levitated samples tend to be embedded mostly within the bulk.

### Viral distribution in sessile samples

**FIG. 4.**
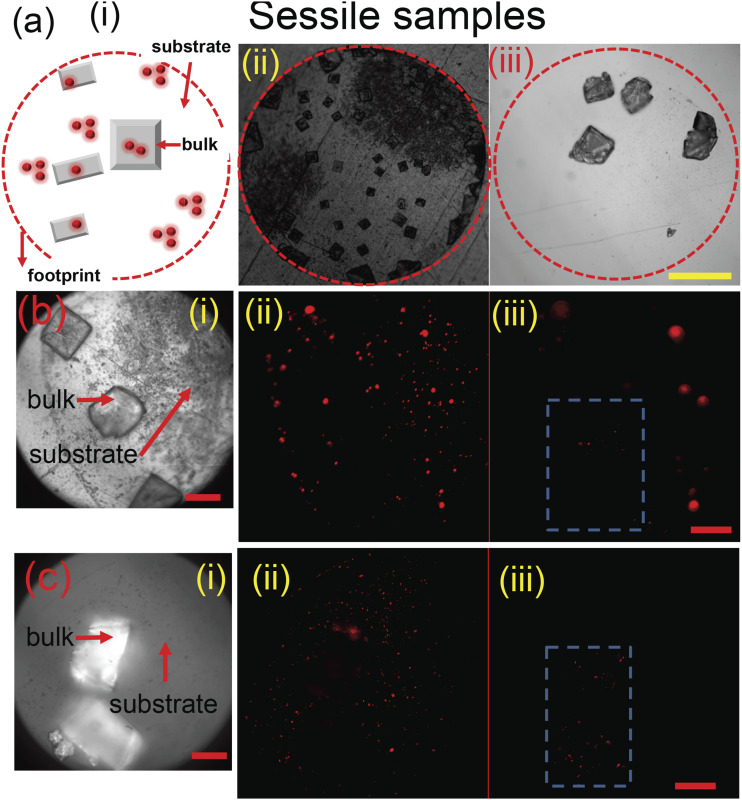
(a) (i) Schematic depicting distribution of nanoparticles within a sessile precipitate. The dashed circle depicts the initial wetted area of the droplet. The complete view of the sessile precipitate on (ii) steel (iii) glass surfaces. (b) (i) The magnified image of the precipitate on the steel surface. Fluorescent images of the same region showing particles exposed on (ii) the substrate and (iii) the bulk of the crystal. (c) The same sequence of images as (b) on the glass surface. Scale bar in yellow equals 0.25 mm and in red equals 40 *µ*m.

The respiratory droplets settle at a rate inversely proportional to the square of their diameters. Thus, droplets as large as *D*_0_ = 550 *μ*m would naturally settle very fast and form fomites. We have used acoustic levitation to mimic much longer residence times of smaller droplets which completely dry while airborne. In order to mimic fomites, droplets of the same volume and particle and salt loading as the levitated droplets were directly dried on steel and glass surfaces. The value of *Pe* ≫ 1 for the sessile case implies that the salt molecules will be under the convective flow field inside. As a result, small crystals of NaCl are distributed across the droplet footprint, as shown in Figs. 4(a–i). The velocity scale for *Pe* calculation is based on $U\approx \frac{r}{t_{f}}$, where $t_{f}\approx \frac{m_{0}}{dm\;/dt}\approx \frac{m_{0}}{\pi rD_{aw}\left(1-RH\right)\rho_{v}f\left(\theta \right)}$, *f*(*θ*) = 0.27*θ*^2^ + 1.3, and the initial value of fomites contact angle *θ* (∼20° on glass and 60° on steel).^22^ Fluorescence images are acquired within the perimeter of the surface precipitate, as shown in Figs. 4(b-i) and 4(c-i) for steel and glass, respectively. The signal from the substrate [Figs. 4(b-ii) and 4(c-ii)] is integrated across the footprint and is denoted as *I*_*substrate*_. Similarly, the integrated intensity from the crystals [Figs. 4(b-iii) and 4(c-ii)] is denoted as *I*_*bulk*_. We define the fraction of fluorescence intensity from exposed particles as $I=\frac{\sum I_{substrate}}{I_{total}}$, where *I*_*total*_ = *I*_*substrate*_ + *I*_*bulk*_. Since the given volume of the droplet contains n ∼ 8 × 10^6^ particles, the average number of particles at the surface is *n*_*exp*_ = *nI*. On glass, *n*_*exp*_ ∼ 5 × 10^6^ while in the case of steel substrates, *n*_*exp*_ ∼ 7 × 10^6^. The variation in the numbers between steel and glass is due to the affinity of the substrate–particle interaction^53^ as well as the internal flow structure.^54^ Nonetheless, precipitates in fomites indeed show a greater percentage of exposed particles (∼80–90%) on the substrates than embedded in crystals as air-borne counterparts. The Probabilistic Analysis for National Threats Hazards and Risks (PANTHR) database^55^ predicts the virus lifetime to be significantly shorter (∼100 times) in air-borne precipitates than those on solid surfaces. This correlates with the presented experimental findings that virions are more exposed in dried settled droplets as opposed to their airborne counterparts.

## CONCLUSIONS

In summary, a nano-colloidal system is successfully used to mimic the evaporation and precipitation dynamics of an isolated mucosalivary droplet. Theoretical and experimental arguments are presented to show how the evaporation leads to salt crystallization, which traps the virion-substitutes at different layers of the air-borne precipitate. We note that the distribution and location of virions in desiccated, precipitated, and crystallized respiratory droplets may affect their long-term survivability since the local environment (e.g., pH level) differs, for example, inside and outside the crystals. Fluorescent microscopy correlates the lower survival rates of viruses in the air-borne precipitates to its lower number of exposed virions, but the relevant biochemistry is beyond the scope of this article. Motility of cellular organisms is well studied^56^ and can be applied to viruses.^26^ However, motility of viruses is not considered here within the droplet drying time. We end this exposition by addressing the future direction. We seek to extend our current study by replacing spherical nanoparticles with structured or functionalized VLPs and, eventually, with live virus particles. The latter will, however, require a BSL2 category laboratory.

## SUPPLEMENTARY MATERIAL

See the supplementary material for additional figures to support the data.

## DATA AVAILABILITY

The data that support the findings of this study are available within the article and its supplementary material.
